# Validation of an Excretory/Secretory Antigen Based-Elisa for the Diagnosis of *Opisthorchis felineus* Infection in Humans from Low Trematode Endemic Areas

**DOI:** 10.1371/journal.pone.0062267

**Published:** 2013-05-09

**Authors:** Maria Angeles Gómez-Morales, Alessandra Ludovisi, Marco Amati, Edoardo Pozio

**Affiliations:** Department of Infectious, Parasitic and Immunomediated Diseases, Istituto Superiore di Sanità, Rome, Italy; Thomas Jefferson University, United States of America

## Abstract

Since opisthorchiasis does not show pathognomonic signs or symptoms, physicians can have serious problems to make a differential diagnosis of this infection in non endemic areas, in particular when there is a simultaneous occurrence with other seasonal infections. Moreover, symptomatic infections due to *O. felineus* can last a few weeks and then the signs and symptoms disappear, but the worms survive in the bile ducts for years causing hepatobiliary diseases including hepatomegaly, cholangitis, fibrosis of the periportal system, cholecystitis, and gallstones. Consequently, an early diagnosis prevents chronicity and loss of working days. The detection of specific antibodies has been considered as a complementary tool to the fecal examination to establish the definitive diagnosis of this infection and for the follow up. Therefore the aim of this work was the development and validation of an enzyme-linked immunosorbent assay (ELISA) using excretory/secretory antigens (ESA) from *O. felineus* adult worms to detect anti-*Opisthorchis* IgG in human sera. A total of 370 human sera were tested: 144 sera from persons with a confirmed diagnosis of opisthorchiasis, 110 sera from healthy Italian people, and 116 sera from people with other parasitic or non-parasitic infections. Results were analyzed by receiver-operator characteristic (ROC) curve analysis. The accuracy of the test, calculated by the area under curve (AUC), yielded a 0.999 value, indicating the high performance of the test. The sensitivity was 100% (95% CI: 97.40% to 100%) and no false-negative sera were detected; the specificity was 99.09% (95% CI: 95.02% to 99.83%). The validated ELISA shows a good performance in terms of sensitivity, repeatability and reproducibility, and it is suitable to detect anti-*Opisthorchis* IgG in human sera for diagnostic purposes and for the follow up to assess the efficacy of drug treatment.

## Introduction

Opisthorchiasis and clonorchiasis are zoonotic diseases caused by liver flukes of the family Opisthorchiidae (*Ophisthorchis viverrini*, *O. felineus* and *Clonorchis sinensis*) which are transmitted by the ingestion of raw or undercooked fish parasitized by the larval stage (metacercaria). Six hundred and eighty million people throughout the world are estimated to be at risk of infection by these parasites, although each species have a particular geographical distribution [1]. *Ophisthorchis felineus* infection has been documented in humans and/or animals in 13 countries of the European Union [2]. In Italy, this parasite was first described in cats and dogs in Tuscany and Piedmont Regions, yet for over 100 years the infection was not detected or reported in humans and no investigation on this pathogen was carried out [3], [4]. This scenario has changed radically in the last decade with the occurrence of several outbreaks of acute human infections [2], [5]–[8].

Liver flukes have a complex biological cycle; they need two intermediate (a freshwater snail and a fish) and one definitive (a fish eating carnivore) hosts to complete their cycle. A wide range of species of freshwater fish of the family Cyprinidae can be naturally infected by these trematodes. Carnivore mammals such as cats, dogs, and foxes act as definitive hosts where the parasite develops into adults in the intra- and extra-hepatic bile ducts and in the gallbladder. Humans are an accidental host [2], [9].

Most people with opistorchiasis or clonorchiasis have unspecific symptoms or no symptoms at all, whereas heavy and long lasting infections are linked to hepatobiliary diseases including hepatomegaly, cholangitis, fibrosis of the periportal system, cholecystitis, and gallstones, and are strongly associated with cholangiocarcinoma (CCA). *O. viverrini* and *C. sinensis* are classified as group 1 carcinogens by the International Agency for Research on Cancer [10], [11]. Regarding *O. felineus*, acute and mild infections with unspecific symptoms and asymptomatic infections have been described, but direct evidences supporting a role of *O. felineus* infection as a risk factor for CCA development are scarce [8], [9], [12].

A specific and early diagnosis of opisthorchiasis in humans is crucial for an appropriate and timely treatment. Since opisthorchiasis does not show pathognomonic signs or symptoms, physicians can have serious problems to make a differential diagnosis of this infection in non endemic areas, in particular when there is a simultaneous occurrence with other seasonal infections, for instance the flu [8]. Moreover, symptomatic infections due to *O. felineus* can last a few weeks and then the signs and symptoms disappear, but the worms survive in the bile ducts for years causing hepatobiliary diseases [8]. The time between infection and the detection of anti-*Opisthorchis* IgG ranges from three to eight weeks [6]–[8]. Consequently, an early diagnosis prevents chronicity, to lose working days, and decreasing the potential risk to develop CCA .

Even if detection of fluke eggs in stools represent the best way to reach a definitive diagnosis of opisthorchiasis, it has become increasingly unreliable in cases of low worm burden [13], [14]. Studies in humans have shown a close relationship between parasite-specific IgG and intensity of *O. viverrini* infection [15]–[17]. Moreover in *O. viverrini* infections, the level of parasite-specific IgG is correlated to the severity of the clinical disease rather than to the egg counts in stools [16], [18]. Consequently, the detection of specific antibodies has been considered as a complementary tool to establish the definitive diagnosis of this infection [19]–[21]. In addition, serology is an excellent tool to monitor the success of the treatment during the follow up [6].

The serodiagnosis of liver fluke infections caused by *O. viverrini* and *C. sinensis* has been attempted using crude adult extracts, metabolic products and egg antigens together with different immunodiagnostic methods, producing results of varying degrees of sensitivity and specificity [16], [17], [19], [22]–[26]. The indirect haemagglutination test, intradermal test and enzyme-linked immunosorbent assay (ELISA), have been developed using the crude somatic extract of *O. felineus* adult worms as antigens [23]. According to Meniavtseva et al., the ELISA shows the best performance among the serological tests [27]. However, its suitability in the detection of *O. felineus* infection in humans is limited by the lack of information on the sensitivity and specificity of the test.

Therefore, the aim of this work was the development and validation of an ELISA using excretory/secretory antigens from *O. felineus* adult worms to detect anti-*Opisthorchis* IgG antibodies in human sera.

## Materials and Methods

### Ethics statement

The human sera have been collected by various Italian health services and have been sent to ISS on different times for a confirmation of the diagnosis of opisthorchiasis or other parasitic infections or as a negative reference population to calculate the cut-off value for serological assays. All samples were received in an anonymous form. Written informed consent was received to all patients who provided serum samples. The present work was approved by the Ethics Committee of the Istituto Superiore di Sanità (ISS, www.iss.it/coet/?lang=1)

Naturally infected tenches (*Tinca tinca*) fished from the Bolsena lake (Viterbo province, Central Italy), were from the local market.

Syrian golden hamsters (*Mesocricetus auratus*) were housed in the Animal Care Unit of the Istituto Superiore di Sanità (ISS) and their use was approved by the Animal Ethics Committee of the ISS. Experiments were performed according to the European Directive 63/2010.

### Antigens

Naturally infected tenches (*Tinca tinca*) from the Bolsena lake (Viterbo province, Central Italy) were skinned and the filets were mechanically chopped and treated with artificial gastric juice (9 g pepsin, powder 1∶10 000 NF, 7 ml 25% HCl in 1 L tap water) using a magnetic stirrer at 38°C. After approximately 30 min, the flesh was digested, the metacercariae were isolated by sedimentation, counted in triplicate, and orally administered (50–60 metacercariae per animal) to 100 g Syrian golden hamsters (*Mesocricetus auratus*) housed in the Animal Care Unit of the Istituto Superiore di Sanità, according to the European Directive 63/2010 [28]. From two months post infection (p.i.) onward, the infection of the hamsters was weekly monitored by the egg count in fecal samples. Four months p.i., animals were euthanized and the liver was removed and washed twice in warm (37°C) phosphate buffered saline (PBS) pH 7.2 in a Petri dish. The gall bladder was opened with small scissors and the enlarged bile ducts were very gently opened to avoid cutting the trematodes. To release the liver flukes from the most remote areas of the organ, a gently pressure was applied to the liver. Liver flukes recovered from the biliary ducts were transferred to 50 mL tubes and washed 5 times in PBS supplemented with Penicillin (500 units/ml) and Streptomycin (500 µg/ml), (GIBCO, Grand Island, NY, USA), at 37°C. Liver flukes (7–12×1.5–2.5 mm) were additionally washed four times allowing them to settle in RPMI 1640 (GIBCO, Grand Island, NY, USA) supplemented with penicillin (500 U/ml) and streptomycin (500 µg/ml).

To prepare excretory/secretory antigens (ESA), 5 worms per 5 mL were suspended in RPMI 1640 supplemented with 1 M HEPES, 200 mML-glutamine, 100 mM Na-pyruvate (all from GIBCO, Grand Island, NY, USA), and 5,000 units of penicillin/streptomycin (Euroclone, BioAir s.r.l., Italy), and incubated with 5% CO_2_ in 6 well culture plates (Corning Life Sciences, Pittsburg, PA, USA) at 37°C for up 15 days. The culture fluid was changed every 24 h, dead worms were removed, pooled, and centrifuged to remove the eggs. The supernatants collected daily were pooled and transferred to 50 mL conical tubes. The supernatants were filtered through a 0.2 µm YM-5 filter, concentrated 100 times in an Amicon® pressure concentrating chamber (Amicon Inc. Billerica, MA, USA), and then dialysed against PBS several times. To determine the protein concentration and to establish the quality of the batch (i.e., no bacterial or somatic contamination), the optical density (OD) was evaluated at a 280/260 nm ratio; antigens with a ratio higher than 1.0 were used. Finally, 1 µL/mg of a cocktail of protein inhibitors (Sigma P8465, Saint Louis, MO, USA) was added to the antigens which were aliquoted and stored at −80°C.

### Human sera

A total of 370 human serum samples were analyzed. Of these, 110 were from healthy Italians, who, according to the Italian law, were considered suitable for blood donation and lived in a non–endemic area for trematode infections (true negative, TN); 144 sera were from people with a confirmed diagnosis of opisthorchiasis based on the presence of *O. felineus* eggs in their stools (true positive, TP). After formol-ether concentration of fecal samples, parasites were searched by light microscopy and, if the samples were negative, *O. felineus* DNA was searched in the fecal sediment by PCR [7]. These people acquired the infection in the course of *O. felineus* outbreaks which occurred in Italy [6]–[8]. All serum samples used in this study originated from adults, about 50% male. Additional 116 serum samples from people with other parasitic or non-parasitic infections were tested: *Toxoplasma gondii* (7), *Cysticercus cellulosae* (14), *Echinococcus granulosus* (10), *Loa Loa* (9), *Toxocara* spp. (15), *Anisakis* spp. (3), *Trichinella* spp (44), *Fasciola hepatica* (10), HIV- *Cryptosporidium* co-infection (3), and HIV-*Enterocytozoon bieneusi* co-infection (1). The diagnosis of these parasitic infections were based on parasitological and/or serological tests. All participants provided oral informed consent to have blood samples drawn.

### ELISA

A standard protocol was used. Briefly, 96-well microtiter plates (Nunc, Roskilde, Denmark) were filled with 100 µl/well of *O. felineus* ESA (2 µg/ml) in carbonate buffered saline pH 9.6±0.2. After incubation at 37°C for 1 h, the plates were washed three times by an automatic plate washer (Dynex Technologies, Denkendorf, Germany) with washing solution (0.5% Tween 20 in PBS, pH 7.3±0.2), blocked by adding 200 µl/well of blocking solution (0.5% BSA, 1% Tween 20), and incubated at 37°C for 1 h. After another washing, 100 µl/well of each 1/200 diluted serum sample was added in duplicate, and the plates were incubated at 37°C for 30 min. After another washing, 100 µl/well of the diluted anti–human IgG peroxidase-labeled antibodies (Kierkegaard and Perry Laboratories, Gaithersburg, MD, USA) were added, and incubated at 37°C for 1 h. Finally, after a last wash, 100 µl/well of the substrate solution containing 3, 3′,5,5′–tetramethylbenzidine and 0.02% of hydrogen peroxide in a citric acid buffer, was added and the plates were incubated at room temperature. The reaction was stopped by adding 50 µl/well of 1 N HCl solution. The OD value was obtained by reading the reaction at 450 nm using an ELISA plate microtiter reader (Dynex Technologies). Each plate contained four TP and four TN reference serum samples, each of which was tested in duplicate. Since raw OD values are absolute measurements that are influenced by test temperature, test parameters, and photometric instruments, the results were expressed as a function of the reactivity of the positive control serum sample with the highest value among the four included in each run of the assay. This control must yield a result that is in the linear range of measurement. The mean OD values of the TP and TN reference sera, as well as the mean OD values of the duplicate test sera, were then calculated, and for each serum sample, an ELISA index (I_E_) expressed as the percentage of positivity was calculated according to the following equation:
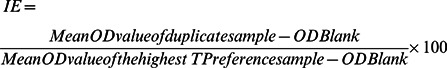



### Statistical analysis

The receiver-operator characteristic (ROC) curve analysis was carried out using the EpiTools epidemiological calculators [29]. This procedure optimizes the interpretation of the ELISA when well-defined positive and negative populations are available for the analysis. The ROC curve is a graphical plot of the sensitivity versus “1 – specificity” for a binary classifier system, using various cut-off values. This allows to select the cut-off value that gives the best balance of sensitivity and specificity for the test under consideration [30]. The repeatability of the test was evaluated by comparing the differences between the OD values of the serum duplicates and their mean [31]. To determine which parameter provided the most accurate result (i.e., the mean OD of the duplicates or the *I_E_*), the area under the ROC curve was calculated for each parameter. The specificity and the sensitivity of the test were interpreted according to the ROC analysis. The interassay variability was assessed by testing three positive and six negative control sera in eight different work sessions, the mean of the OD values was determined and used to calculate the coefficient of variation (CV).

### Accreditation

The laboratory is accredited according to ISO/IEC 17025:2005 by the Italian accreditation body ACCREDIA (www.accredia.it).

## Results

**Figure 1:**
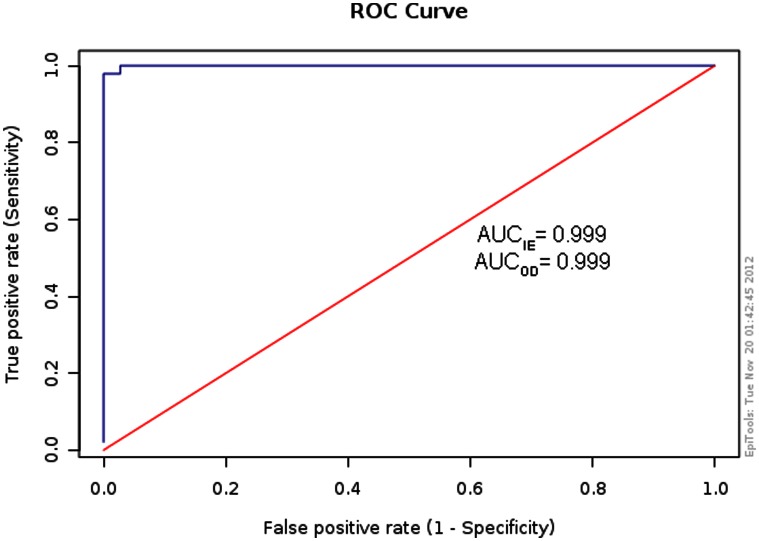
The receiver-operator characteristic (ROC) curve. The ROC curve was built for 110 sera from healthy blood donors and 144 sera from people with confirmed opisthorchiasis. The area under the ROC curve (accuracy) was determined for the ELISA index (I_E_), expressed as percentage of positivity (see text), and for the OD mean of duplicates, yielding 0.999 in both cases.

ROC curves were built with data from the two defined TP and TN reference populations. The descriptive statistical summary of the data is shown in Table 1. The AUC was 0.999 for both the mean OD value of the duplicates and the I_E_, indicating that the two parameters provided equally accurate results (Fig. 1). The ROC optimized cut-offs were 18% for I_E_ and 0.341 for the OD values; on the basis of these cut-off values, the sensitivity reached 100% (95% CI: 97.40% to 100%) and the specificity yielded 99.09% (95% CI: 95.02% to 99.83%). No false-negative (FN) sera were found; however, 5 (4.5%) false positive (FP) sera were detected among TN sera and 17 (14.6%) FP sera were detected from persons with other parasitic infections, namely one with *C. cellulose*, three with *E. granulosus*, four with *L. loa*, one with *Toxocara* spp., and eight with *Trichinella* spp.

**Table 1:** Descriptive indices of ELISA results to detect anti-*Opisthorchis* IgG in sera from persons with a confirmed diagnosis of opisthorchiasis (infected), and from healthy persons (non-infected).

Statistical indices	Infected (144)		Non-infected (110)	
	OD	I_E_	OD	I_E_
Mean	1.22	86.5	0.128	9.03
SD	0.385	39.4	0.061	3.99
p50	1.19	72.6	0.122	8.61
p25	0.937	58.2	0.085	6.33
p75	1.51	108	0.157	10.9
Minimum	0.341	18	0.021	1.51
Maximum	2. 29	171	0.349	25.7

The diagnostic specificity and sensitivity of the ELISA, including serum samples from people with other parasitic or non-parasitic infections, are summarized in Table 2. The sensitivity of the test was 100% (95% CI: 97.45% to 100%) and the specificity dropped to 90.27% (95% CI: 85.63% to 93.80%) with a positive predictive value of 86.75% (95% CI: 80.62–91.50%) and a negative predictive value of 100% (95% CI: 98.19–100%)

**Table 2:** Diagnostic sensitivity, specificity, and positive and negative predictive values (cut off 17%, 95% CI) for serum samples from people with a diagnosis of *Opisthorchis felineus* infection confirmed by the detection of *O. felineus* eggs in their stool, serum samples from *Opisthorchis* sp. -free people, and serum samples from people suffering from other parasitic or non-parasitic infections.^a^

	*Opisthorchis* infection				
ELISA result	Infected	n	Uninfected	n	Total
Positive	True positive	144	False positive	22	166
Negative	False negative	0	True negative	204	204
Total		144		226	

a Sensitivity = 100% (95% CI: 97.45–100%), it was calculated as TP/(TP+FN); Specificity = 90.27% (95% CI: 85.63–93.80%), it was calculated as TN/(TN+FP);

c Positive predictive value = 86.75% (95% CI: 80.62–91.50%), it was calculated as TP/(TP+FP); Negative predictive value = 100% (95% CI: 98.19–100%), it was calculated as TN/(TN+FN). TP, TN, FP, and FN are the numbers of true positives, true negatives, false positives, and false negatives, respectively.

**Figure 2:**
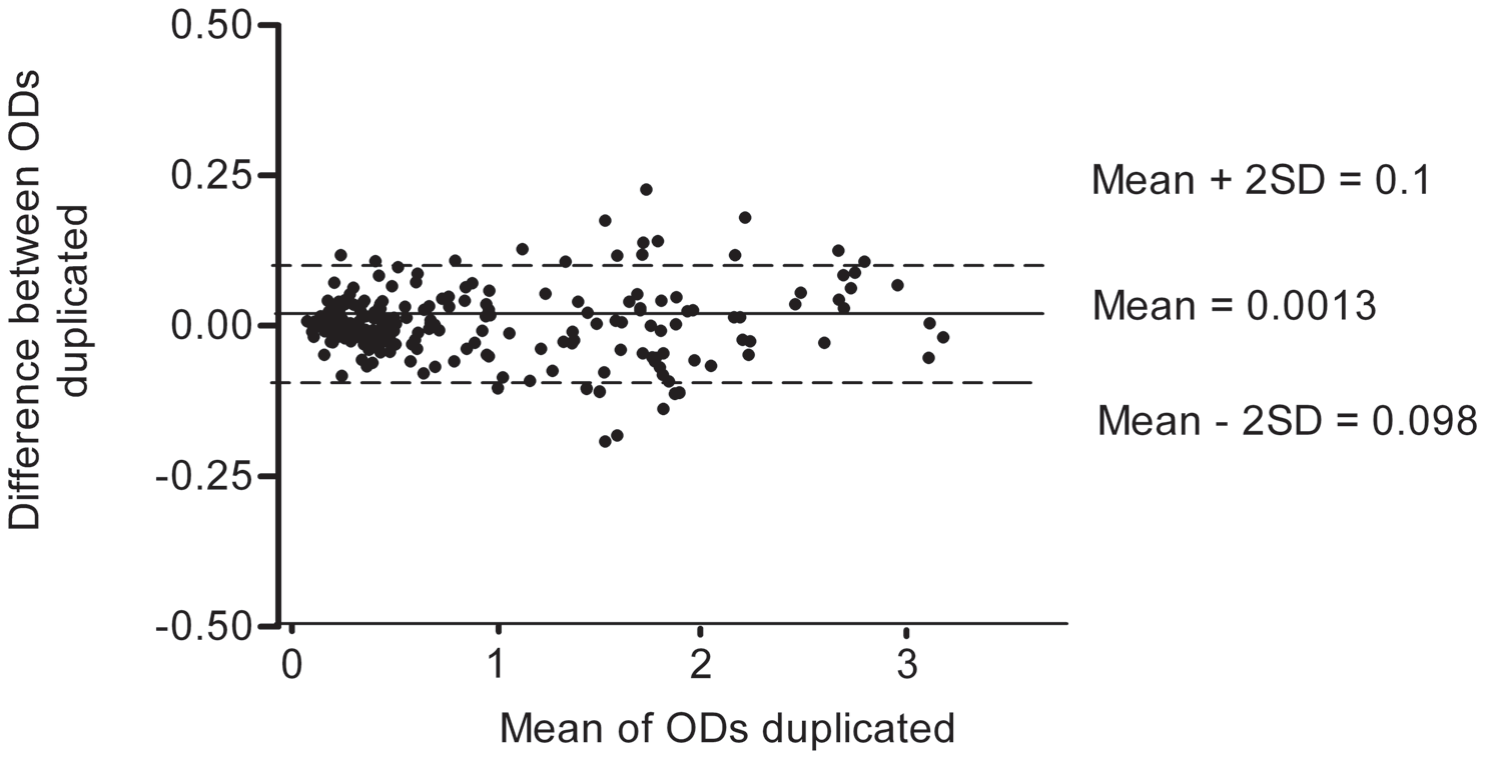
Scatter plot of the differences between the optical density (OD) of the serum duplicates and their means. OD was recorded at 450 nm.

The interassay variability was assessed by calculating the CV with data from eight different working sessions, and did not exceed 10% and 20% for the three positive and the six negative serum samples, respectively (Table 3). Regarding repeatability, no trend was found to increase OD differences between duplicates as their means increase (Figure 2).

**Table 3:** ELISA reproducibility.

Indices	Serum samples								
	1	2	3	4	5	6	7	8	9
I_E_ mean	97	58.6	77.4	4.3	11.9	12.9	10.5	10.5	14.4
SD	4.3	4.8	7.0	0.9	1.8	1.2	1.0	1.3	0.8
CV (%)	5	8.1	9.0	6.5	10.9	10.4	7.9	19.0	6.1

Data from three *Opisthorchis*-positive (N. 1–3) and six *Opisthorchis*-negative (N. 4–9) serum samples corresponding to eight different working sessions.

## Discussion

In the field of the diagnosis for human parasitic diseases, there is an urgent need to validate serological tests and to show their performance in terms of sensitivity, specificity, accuracy, and reproducibility. Ideally, the test results should be combined with other laboratory findings, and with clinical and epidemiological data to provide a reliable diagnosis of the infection. It is necessary to keep in mind that serological methods cannot replace the direct microscopic examination of the stool and the identification of the *O. felineus* eggs. However, the parasitological diagnosis alone has several drawbacks: 1) only an experienced microscopist can make an accurate diagnosis on fecal samples due to the small egg size (22–32×10–22 µm) and their peculiar morphology; 2) there is a considerable percentage of sero-positive cases identified among *Opisthorchis* sp. egg-negative individuals with a pauci-symptomatic clinical disease or with a delayed diagnosis [6]–[8], [32], or patients with a biliary obstruction in whom eggs cannot be recovered in the feces [21]. Consequently, serology is very important for the diagnosis of liver fluke infections [6], [8], [9], [19]–[21] and may offer help in the follow-up to assess the efficacy of drug treatment as shown by Traverso et al. [6] and Armignacco et al. [8]. Several methods have been used for the serological diagnosis of *O. felineus* in humans [23], [27], [33], [34]. However, as far as we know, no serological test has been validated using a large panel of sera from people with confirmed opisthorchiasis due to *O. felineus*, healthy people, or people with health disorders unrelated to opisthorchiasis.

A central point in the validation of a serological test is the determination of the cut-off; therefore, the sample size must be large enough to minimize the stochastic uncertainty in the cut-off selection [29]. Furthermore, positive and negative reference populations should be properly defined. The high sensitivity showed by the developed ELISA (100%, CI 95% CI: 97.40% to 100%), yielding no FN results, can be explained by the use of ESA. This is in agreement with the results of immunolocalization studies which showed that the surface structures of *O. felineus* stimulate a low B-cell immune response in comparison with the structures of the excretory-secretory system of the parasite and their products, which contain a lot of antigens able to induce a strong B-immune response in humans [35].

On the other hand, ESA affects the test specificity. In fact, the specificity of the developed ELISA dropped to 90.31% (95% CI: 85.69% to 93.82%) when sera from people with other health disorders unrelated to *O. felineus* are included, since 17 (14.6%) false-positive reactions were detected among them. Moreover, 5 FP were observed among TN sera (Table 2). A main problem in the serological diagnosis of parasitic infections, and especially for those caused by helminths, is the cross-reactivity, in particular when parasite crude extracts (CE) are used. In fact, using CE from adults, metacercarie and eggs, and ESA from adults, some authors have stated that the specificity in the detection of circulating antibodies to *O. viverrini* is limited by the cross-reactive nature of the antigens [17], [19], [22], [24], [25], [36]–[41]. This is particularly important in developing countries where people are infected with liver and intestinal flukes, with other helminths, and with protozoa [17]. In the course of *C. sinensis* infections, the serological diagnosis by ELISA yielded high sensitivity (80%) but low specificity (33.3%) in high risk groups with a history of raw fish consumption. It follows that this diagnostic test is not suitable for epidemiological surveys in developing countries where people are frequently exposed to other parasitic infections [42]. Attempts to obtain more specific *O. viverrini* antigens have been done by the partial purification of fractions from adult and egg crude extracts as well as from ESA [11], [20], [24], [26], [41].

For *O. felineus* human infections, information regarding the specificity of serological tests for diagnostic purposes is scarce [9], [12]. “ESA” is a working definition, with an unclear distinction between products actively exported through secretory pathways and those that may be diffused or that may leak from the parasite soma. ESA consists of a number of compounds, including glycans, lipids and enzymes, some of which are shared with other helminths [43]. According to Glupov et al. and Kotelkin et al., ESA components of molecular weight of 105, 74, and 70 Kd may have a potential use for a more specific immunodiagnosis [44], [45]. Recently, a cathepsin B-like cysteine protease belonging to family C1 present in the *O. viverrini* ESA, has been cloned, expressed, and used in an ELISA to detect specific IgG in sera from persons from an endemic area. This ELISA yielded sensitivity and a specificity of 67% and 81%, respectively [46]. In the present work, the validated ELISA, that used complete ESA, reaches 100% sensibility and 90.31% specificity. The difference in specificity between our ELISA and that developed by Sripa et al. could be due to the two target populations characterized by a high prevalence for liver and intestinal flukes in Thailand and by the absence of trematode infections in most of the Italian territory [46].

In the developed ELISA, more than 95% of the differences between OD values of serum duplicates are less than two standard deviations; it follows that the ability to reproduce ELISA is high [31]. Furthermore, the interassay variability test, based on data from four sera in eight working sessions, was only 10% for the positive sera and did not exceed 20% for the negative sera, indicating a good reproducibility of the ELISA (Table 3).

The validated ELISA shows an excellent performance in terms of sensitivity, repeatability, and reproducibility, and an acceptable specificity. Therefore, we conclude that the method is suitable for detecting anti-*O. felineus* antibodies in human sera, mainly for diagnostic purposes in association with epidemiological and clinical data.
